# Delayed Reconstruction by Total Calcaneal Allograft following Calcanectomy: Is It an Option?

**DOI:** 10.1155/2016/4012180

**Published:** 2016-11-20

**Authors:** Benjamin Degeorge, Louis Dagneaux, David Forget, Florent Gaillard, François Canovas

**Affiliations:** Department of Orthopedic Surgery, Division of Lower Limb Surgery, Lapeyronie University Hospital, 371 Avenue du Doyen Gaston Giraud, 34295 Montpellier Cedex 5, France

## Abstract

Many options are available in literature for the management of delayed reconstruction following calcanectomy. In cases of low-grade tumor lesions, conservative surgery can be considered. We describe a case of delayed reconstruction by calcaneal allograft after calcanectomy for low-grade chondrosarcoma. At 12-month follow-up, the patient had no pain; MSTS score and AOFAS score were satisfactory. Subtalar nonunion was observed with no secondary displacement or graft necrosis. The aim of conservative treatment for this patient was to restore normal gait with plantigrade locomotion and function of the Achilles tendon. Calcaneal reconstruction by total allograft is an alternative approach following calcanectomy for calcaneal tumors. We also discussed other options of calcaneal reconstruction.

## 1. Introduction 

Delayed reconstruction is needed in rare cases, especially following calcanectomy. For example, conservative surgery can be considered in cases of low-grade tumors. The aim of this report was to make a functional assessment of delayed reconstruction of the calcaneus by total allograft and to discuss alternative treatments.

## 2. Clinical Case 

**Figure 1:**
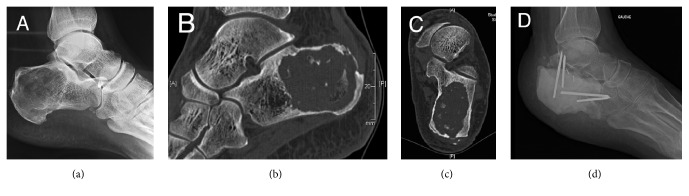
Clinical history: X-ray (a) and CT-scan (b and c) images of calcaneal chondrosarcoma showing a heterogeneous, lytic picture with intracystic calcifications. Visualization of a cortical rupture of the greater tuberosity with involvement of the Achilles tendon. Lateral X-ray (d) showing the spacer following calcanectomy with talocalcaneal and calcaneocuboid fixation.

A 58-year-old patient was referred to our University Hospital in June 2014 for a chronic wound of the left heel. Review of the patient's clinical history revealed total calcanectomy in 2007 with a cement spacer fixed by pins after bone cancer. Pathological examination showed a well-differentiated cartilaginous tumor with bone resorption and hyaline tumor matrix without myxoid reshuffle. Investigations were compatible with a low grade of calcaneal chondrosarcoma with involvement of the Achilles tendon (Figure 1).

**Figure 2:**
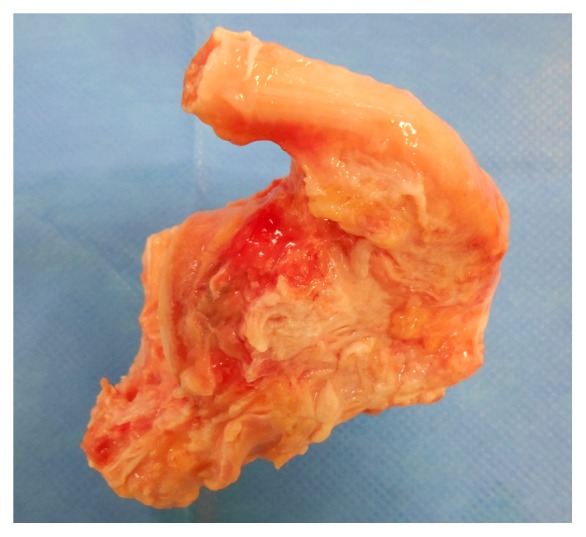
Photograph of the total calcaneal allograft with the distal extremity of the Achilles tendon.

**Figure 3:**
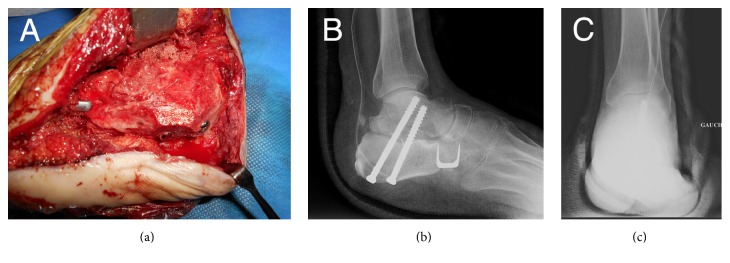
Intraoperative lateral view of the calcaneal allograft arthrodesis (a) and postoperative X-ray examination (b and c) of the calcaneal allograft and double arthrodesis.

In January 2015, delayed allograft bone reconstruction was performed using total calcaneus with the distal extremity of the Achilles tendon (Figure 2) retrieved during multiorgan removal and processed in the standard manner. Surgeon performed lateral approach of the calcaneus, avoiding the sural nerve and fibular tendons. After the spacer was extracted by fragmentation, bone scissors were used for joint cartilage removal. The calcaneus allograft was then calibrated with an oscillating saw to obtain a size appropriate for the morphology of the hindfoot. The graft was temporarily fixed by pins under scopic guidance. Double arthrodesis was then performed after spongy bone grafting from the iliac bone: subtalar arthrodesis with two screws of 6.5 mm diameter and calcaneocuboid arthrodesis with a Blount's staple (Figure 3). The plantar fascia and the extremities of the Achilles tendon were sutured at their respective insertion sites on the allograft with a Krackow-type suture using nonabsorbable suture PremiCron® USP 5 after removal. The Achilles peritendon was then sutured to itself to promote its vascularization. Postoperative recommendations were total rest for 3 months followed by gradual resumption of foot contact with the ground in a shoe with heel support. The patient started to walk on the full sole of the foot as from the 4th month, with the aid of two crutches.

**Figure 4:**
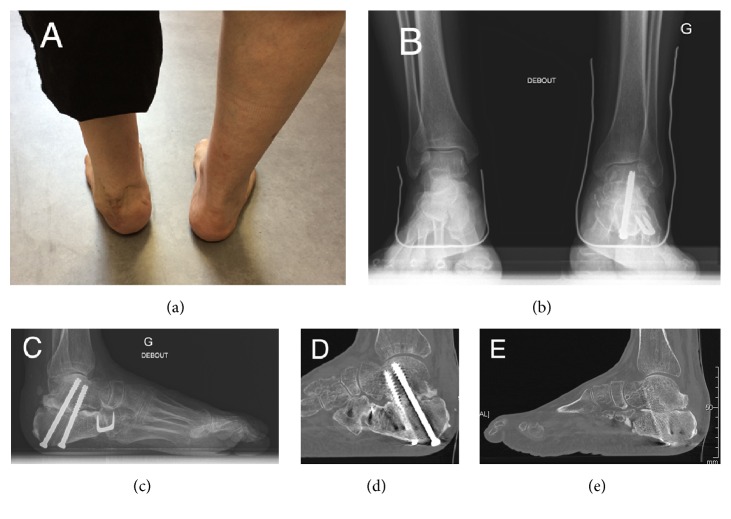
Latest follow-up assessment: Photograph shows a slight varus of the hindfoot (a). X-ray (b) and scan (c) assessment at 12-month follow-up with Meary incidence showing the residual varus of the hindfoot.

At 12-month follow-up there were no signs of tumor relapse. The patient was pain-free and had returned to work (Figure 4), with an MSTS 93 score of 67% and an AOFAS score of 72 points. Dorsiflexion and plantar flexion were 15 and 30 degrees, respectively. Achilles tendon action was normal with muscle strength of 5/5, corresponding to similar contraction of the active plantar flexion compared to the contralateral side and a rise heel position allowed in single leg stance. Testing of subtalar and Chopart joints was painless. Podoscopic examination showed a hindfoot varus and defective medial support. The patient was able to walk barefoot without pain. He was prescribed pronation insoles for daily use over a walking distance of 500 m. X-rays showed a calcaneal varus of few degrees from Meary's method in weight-bearing and CT-scan highlighted a subtalar nonunion (Figure 4). The calcaneocuboid arthrodesis was healed. There was no evidence of secondary displacement, fracture, or graft necrosis.

## 3. Discussion

Chondrosarcomas develop very slowly in the young adult with no overt symptoms. A study by the Mayo Clinic reported a survival rate of 89% at 10-year follow-up [1] despite metastatic evolution in a quarter of the cases. The reference treatment is a surgical resection with satisfactory results. However, treatment by conservative surgery is not restricted to removal of the tumor in free margins [2], and the final aim is to restore normal gait. This involves several factors including bearing of weight without deformation of the hindfoot and normal movement of the Achilles tendon and plantar fascia to allow plantigrade locomotion. Our patient underwent delayed reconstruction 6 years after calcanectomy. There are few documented reports of the surgical procedure and approaches differ between authors (Table 1).

- Ottolenghi and Petracchi [3] and Muscolo et al. [4] were the first to study the possibility of a total calcaneal allograft. In both reports, osteointegration was successful with satisfactory functional results. However, the authors reported secondary osteonecrosis of the hindfoot at 4-year follow-up in both studies.
- Li et al. [2, 5, 6] recommended the use of composite fibular flaps with or without allograft and achieved satisfactory functional and oncologic results. No information was given on postoperative foot statics.
- Scoccianti et al. [7] and Kurvin et al. [8] used vascularized iliac crest bone graft. Owing to its greater volume and according to the size of the resection, the iliac bone graft allowed full weight-bearing and good-quality tissue for arthrodesis. However, the use of free flaps required microsurgical anastomosis including its complications. One case of bone graft fracture was observed in follow-up but without long-term functional consequences.
- Imanishi and Choong [9] and Chou and Malawer [10] used a titan prosthesis after scan planning. Postoperative progress was similar to that following allograft, with successful functional recovery.

Each technique had its specific problems with regard to fixation, soft tissue coverage, donor site morbidity, and functional recovery (Table 2). The use of Chopart's fixation was debatable. To our knowledge, there have been no biomechanical studies of the mode of fixation in calcaneal allografts. We attached the calcaneal allograft by double arthrodesis avoiding the talonavicular joint and using spongy autograft from the iliac bone [11]. The authors dealt with different fixation regarding the type of reconstruction: subtalar fixation [2, 5, 6] or double arthrodesis [4, 8]. Calcaneal prostheses [9, 10] were stable after ligament fixation and without bone fixation.

Soft tissue coverage is not always necessary and the decision to use flaps depends on tumor invasion. Several authors recommend the use of mixed flaps (pediculated fibular [2, 5, 6] or free iliac [7, 8]) for coverage. However, in more than a third of cases repeat surgery was required for local complications. Despite a reported success rate of 96%, microvascular anastomoses of the free flaps lead to further complications [12]. The use of flaps may be restricted by problems of tissue autonomization following calcaneal prosthesis. In calcaneal allografts, it is possible to include a sural pediculated flap and maintain epicritic plantar sensitivity.

Total calcaneal allograft is an alternative treatment of low-grade calcaneal tumors. We describe its use in delayed construction by allograft following calcanectomy. At 12-month follow-up, our patient had satisfactory clinical and functional scores. However, long-term monitoring is required to assess allograft survival in this indication.

## Figures and Tables

**Table 1:** Review of the literature of the different options of reconstruction following calcanectomy.

Authors	Date	NC	Surgery	Characteristics	MSTS (%)	AOFAS	FU (y)
Imanishi and Choong [9]	2015	1	Calcaneal prosthesis	No tumor recurrence	/	82	0.4
Li and Wang [5]	2014	5-4	Allograft + pediculated composite fibular flap versus amputation	No local tumor recurrence	74–83	/	3.5
Li et al. [2]	2012	4	Allograft + pediculated composite fibular flap	2 local repeated surgeries No tumor recurrence	93	80–95	2
Li et al. [6]	2010	5	Pediculated composite fibular flap	2 local repeated surgeries No tumor recurrence	93	80–95	4.2
Scoccianti et al. [7]	2009	2	Free composite iliac flap	1 fracture No tumor recurrence	/	/	7.1
Kurvin et al. [8]	2008	1	Free composite iliac flap	/	/	/	2.6
Chou and Malawer [10]	2007	1	Calcaneal prosthesis	No tumor recurrence	/	67	12
Muscolo et al. [4] Ottolenghi and Petracchi [3]	2000	2	Calcaneal autograft + iliac autograft	1 osteonecrosis	/	/	9–32

NC: number of cases; MSTS: Musculoskeletal Tumor Society; AOFAS: American Orthopedic Foot and Ankle Society; FU: follow-up; y: years.

**Table 2:** Comparison of different reconstruction techniques following calcanectomy.

	Fixation	AT suture	Donor site morbidity	Foot statics	Complication at last follow-up (years)	Possibility of soft tissue coverage
Calcaneal allograft [3, 4]	Double arthrodesis	Yes	/	Restored	Osteonecrosis of the graft (32 and 9)	Yes^*∗*^
Composite fibular flap [6]	Arthrodesis ST	No	None in the study Risk of lesions common PN, pain	Restored but strait calcaneal support	3 repeat flaps (4,2)	Yes
Allograft + pediculated composite fibular flap [2, 5]	Arthrodesis AT suture	Yes	None in the study Risk of lesions common PN, pain	Restored	2 repeat flaps (2 and 3,5)	Yes
Free composite iliac flap [7, 8]	Double arthrodesis (ST, CC, and TN)	Yes	Pain Scar	Restored Heel numbness	Graft fracture (7,1 and 2,6)	Yes
Calcaneal prosthesis [9, 10]	ST and CC avivement	Yes + plantar fascia and spring ligament	/	Restored	None (0,4 and 12)	To be assessed

ST: subtalar; CC: calcaneocuboid; AT: Achilles tendon; TN: talonavicular; PN: peroneal nerve; ^*∗*^associated or secondary sural flap.

## References

[1] Itälä A., Leerapun T., Inwards C., Collins M., Scully S. P. (2005). An institutional review of clear cell chondrosarcoma. *Clinical Orthopaedics and Related Research*.

[2] Li J., Pei G., Wang Z., Guo Z., Yang M., Chen G. (2012). Composite biological reconstruction following total calcanectomy of primary calcaneal tumors. *Journal of Surgical Oncology*.

[3] Ottolenghi C. E., Petracchi L. J. (1953). Chondromyxosarcoma of the calcaneus; report of a case of total replacement of involved bone with a homogenous refrigerated calcaneus. *The Journal of Bone and Joint Surgery*.

[4] Muscolo D. L., Ayerza M. A., Aponte-Tinao L. A. (2000). Long-term results of allograft replacement after total calcanectomy. A report of two cases. *The Journal of Bone & Joint Surgery—American Volume*.

[5] Li J., Wang Z. (2014). Surgical treatment of malignant tumors of the calcaneus. *Journal of the American Podiatric Medical Association*.

[6] Li J., Guo Z., Pei G.-X., Wang Z., Chen G.-J., Wu Z.-G. (2010). Limb salvage surgery for calcaneal malignancy. *Journal of Surgical Oncology*.

[7] Scoccianti G., Campanacci D. A., Innocenti M., Beltrami G., Capanna R. (2009). Total calcanectomy and reconstruction with vascularized iliac bone graft for osteoblastoma: a report of two cases. *Foot and Ankle International*.

[8] Kurvin L. A., Volkering C., Keßler S. B. (2008). Calcaneus replacement after total calcanectomy via vascularized pelvis bone. *Foot and Ankle Surgery*.

[9] Imanishi J., Choong P. F. M. (2015). Three-dimensional printed calcaneal prosthesis following total calcanectomy. *International Journal of Surgery Case Reports*.

[10] Chou L. B., Malawer M. M. (2007). Osteosarcoma of the calcaneus treated with prosthetic replacement with twelve years of followup: a case report. *Foot and Ankle International*.

[11] Lareau C. R., Deren M. E., Fantry A., Donahue R. M. J., DiGiovanni C. W. (2015). Does autogenous bone graft work? A logistic regression analysis of data from 159 papers in the foot and ankle literature. *Foot and Ankle Surgery*.

[12] Gao R., Loo S. (2011). Review of 100 consecutive microvascular free flaps. *New Zealand Medical Journal*.

